# Shape Memory Investigation of α-Keratin Fibers as Multi-Coupled Stimuli of Responsive Smart Materials

**DOI:** 10.3390/polym9030087

**Published:** 2017-03-03

**Authors:** Xueliang Xiao, Jinlian Hu, Xiaoting Gui, Kun Qian

**Affiliations:** 1 Institute of Textiles and Clothing, the Hong Kong Polytechnic University, Hong Kong, China; xiao_xueliang@163.com (X.X.); guixiaoting1992@gmail.com (X.G.); 2 Key Laboratory of Eco-Textiles, Ministry of Education, Jiangnan University, Wuxi 214122, China; qiankun_8@163.com

**Keywords:** α-keratin fiber, coupled stimuli, shape memory, network model

## Abstract

Like the water responsive shape memory (SM) effect of β-keratin bird feathers, α-keratin hairs either existing broadly in nature are found responsive to many types of coupled stimuli in SM behaviors. In this article, α-keratin hairs were investigated for the combined stimuli of thermo-solvent, solvent-solvent, and UV (radiation)-reductant sensitive SM abilities. The related netpoints and switches from the hair molecular networks were identified. The experimental results showed that α-keratin hairs manifested a higher ability of shape fixation under thermal stimulus followed with the stimuli of solvent and UV-radiation. Shape recovery from the hair with a temporarily fixed shape showed a higher recovery ability using solvent than the stimuli of heat and UV-radiation. The effects of coupled stimuli on hair’s shape fixation and recovery and on variations of the crystal, disulfide, and hydrogen bonds were studied systematically. A structural network model was thereafter proposed to interpret the multi-coupled stimuli sensitive SM of α-keratin hair. This original study is expected to provide inspiration for exploring other natural fibers to reveal related smart functions and for making more types of remarkable adapted synthetic materials.

## 1. Introduction

A shape memory polymer (SMP) is a material that ‘remembers’ its original shape and that enables the deformed shape to return to its pre-deformed state when stimulated. SMP is usually lightweight, flexible, stretchable, low cost, and easily processible. It belongs to the smart material that associates closely with the changes of a macromolecular network, reversible covalent bonds, and the crystalline phase, e.g., glass transition ($T_{g}$), disulfide bond, and crystal for melting ($T_{m}$) [1]. This endows SMP with a wide range of stimuli that can be conveniently tailored for specific applications. To date, many synthesized SMPs have been reported [2,3,4,5] to adapt to various types of environmental stimuli; for example, temperature, moisture, water, light, electricity, etc. Most SMPs [6,7,8,9,10] are single stimulus responsive to hybrid or co-block synthetic polymers; for example, moisture-sensitive SMPs that are hybridized with chitosan, cupric sulphate pentahydrate, or poly(vinyl alcohol), respectively, that contain a large amount of hydroxyl groups or co-block synthesizing polyhedral oligomeric silsesquioxane molecules and poly(ethylene glycol), for hard and soft segments separately. The shape memory effect (SME) of each SMP is disclosed due to the related lock/unlock effect by external stimulus on the reversible switch of polymeric material, thus varying the flexibility of some parts of the macromolecule chains during shape fixation and recovery [11,12]. In this respect, the reversible switch and invariable structural ingredient sound compulsory in SMP compounds. 

**Figure 1:**
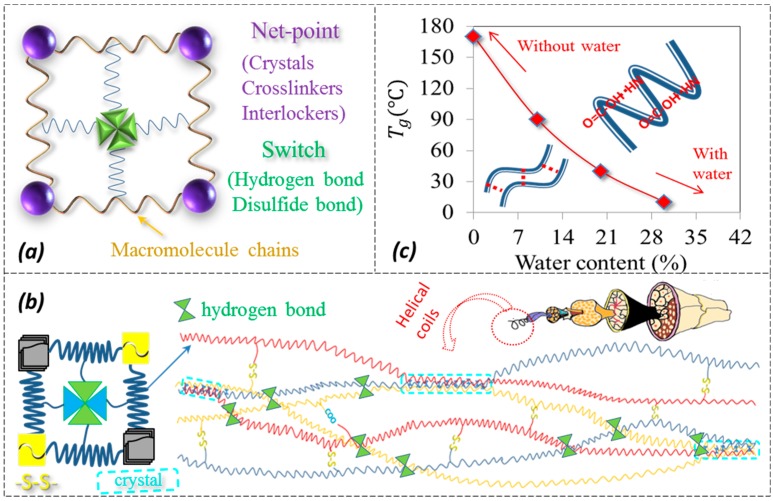
Schematic illustrations of (**a**) a typical netpoint-switch model for shape memory polymers (SMPs) in which candidates for netpoint may be crystals, chemical crosslinkers, or interlockers and for switches may be hydrogen bonds (HBs) or reversible covalent bonds; (**b**) a structural model for α-keratin fiber with discrete crystalline phase linked by α-keratin backbones embedded in an amorphous matrix that contains HBs, disulfide bonds, and salt linkages; the inset shows a twin-netpoint-single-switch model for α-keratin fiber; (**c**) the relationship of wool’s glass transition temperature ($T_{g}$) and the water content for which the inset shows the HBs at intra- and inter-molecule chains for the cause of $T_{g}$ variation with and without water molecules.

In SMP, the invariable structural ingredient, namely netpoint, is stable and may be chemical/physical cross-linkers, a crystalline phase, or an interlocked supramolecular complex [4]. The netpoints connect polymer macromolecular chains in the amorphous region to form a molecular net. It was reported that the entropic elasticity of the net provides the driving force for shape recovery [13]. On the other hand, the role of an SMP’s switch is to lock/unlock the polymer’s temporary shape during the fixation and recovery under particular processes [14]. To design and synthesize a SMP, a recovery force from the netpoints and a locking force from the switch are a necessity. A classical model for an SMP’s compositional structure is shown in Figure 1a, which demonstrates the design rule for synthetic SMPs. In the past decade, many synthetic bio-inspired SMPs and SMP finishing agents have been developed according to the model [15,16,17]. These materials showed strong SME to be responsive to single specific stimuli in fixation and recovery.

Alternatively, in an analogy of structural components to synthetic SMPs, many natural polymers, in particular protein-type biopolymers, are capable of undergoing intelligent adaptations and responses to environmental stimuli through a long history of evolution/selection under ecological conditions [18,19,20,21,22]. For example, recently, peacock tail convert feathers and spider silk, both containing β-folded protein molecular chains, were reported to have obvious water-sensitive SME [18,23]. Referring to α-keratin animal hairs, an evident indication has shown them to have purely four types of stimulus-induced SME perspective [24,25]. In textiles, silks and animal hairs both show higher moisture regain values (10%~15%), indicating a large amount of hydrophilic/polar groups in either intra-molecules or inter-molecules of fiber compared with cellulose-type fibers [26,27]. This indicates that the polar solvents may be good stimuli for such fibers in shape fixation and recovery.

Regarding the α-keratin hair, the internal α-helical folded structure was formed by hydrogen bonds (HBs) between amide N–H and carboxylic C=O groups of successive turns of each helix [28], and the hair matrix [29] was found to be rich in high-sulfur forming glycine tyrosine and cystine proteins. Under dry status, it was reported that the crystalline phase of α-keratin hair fiber (e.g., wool) accounts for 25%~30% of the whole fiber volume indicated by infrared and XRD data [30]. Under wet status, the high-sulfur protein, high-glycine tyrosine proteins, non-helical water penetrable materials, and the absorbed water in hair are all considered an amorphous matrix. A model [31] with a discrete crystalline phase, as shown in Figure 1b, was proposed for α-keratin hair along the hair axis, for which its tensile-relaxation behavior under stretching can be interpreted well. Based on the discrete crystal/matrix model, it was learned that the $T_{g}$ of α-keratin fiber is decreased with an increase of absorbed water for the cause of disruption of HBs between the intra- and inter-macromolecule chains by aqueous molecules [32], as shown in Figure 1c. This results in the easier mobility of unlocked macromolecular chains in the amorphous area under the intact network. The residual groups of the collapsed HBs can be re-formed through the removal of water when α-keratin fiber is dried [33], which indicates the reversible HBs as the switch through the transformation of unlocked/locked status with and without water, alternatively.

Although most synthetic SMPs are sensitive to a single stimulus, a coupled action of two or more types of external stimuli on SMPs sounds more realistic and is of more interest in the industry. For example, the creases of a cotton shirt and a wool suit generated by long-time stacking under wet conditions can be removed by ironing. Here, the stimuli of moisture and heat are performed sequentially on the cotton/wool fabrics for temporary shape fixation and recovery. In addition, coupled factors are usually used to process the α-keratin fibers. For instance, wool yarns and fabrics can be set into the desired shapes permanently under specific temperatures and moistures [34,35,36]. The degree of permanent set depends on the exerted stimuli of heating and humidity on wool fabrics. The reported coupled stimuli of temperature and moisture for the permanent shape set of a wool shirt should be higher than 100 °C and 34% relative humidity (RH) normally [34]. The degree of permanent set increases with the increase of temperature and moisture [36]; thus, the temporary shape set was suggested to be below 100 °C, and the shape could be released in water at room temperature.

Inspired from the multi-responsive stimuli of animal hairs, this article, based on the SME mechanism model of SMP and permanent shape set study of wool, attempts to understand many types of coupled factors on the SME mechanisms of α-keratin fiber in setting temporary shape and shape recovery simultaneously. The effect of coupled factors on related netpoints and switches in the molecular scale are characterized and identified accordingly.

## 2. Materials and Methods

### 2.1. Preparation of α-Keratin Hair Fibers

Wool has been regarded as traditional α-keratin fiber in textiles [29]; however, wool is too fine to demonstrate SME for the naked eye. For the sake of a demonstration, guard hairs (hair diameter > 50 μm) were selected as α-keratin samples. Thus, due to the similarity of inner components and the protein structure with wool, goat hair (rich of guard hairs) was selected to investigate the coupled stimuli of SME of α-keratin fiber. The raw goat guard hairs from an adult goat’s back were purchased from Dite Cashmere Industry Co., Ltd., Nan-gong, Hebei, China. The hair samples were firstly combed to remove the mud and impurities. Then they were screened to select narrow ranges of diameter (80~85 μm) and length (90~100 cm) for the samples for experimental study because most goat guard hairs were found to fall in these range intervals. This selection can ensure the later uniform performance that was obtained from the testing. The samples were then soaked in the chloroform solution for one minute to remove the surface fatty film [37], which was assumed to have a negligible effect on the fiber inner cortex and medulla. The removal of the fatty film could be condusive to an efficient interaction of the hair cortex with different stimuli in the later test. Then, the fiber samples were rinsed twice with distilled water, and they were dried in a 40 °C atmosphere. The moisture regain of the goat hair fibers was measured in the range of 10% and 14%.

### 2.2. Characterization of Fiber Coupled Stimuli of SME 

The conditioned straight keratin fibers were manually wrapped on a circular glass bar, and the shape was maintained in a humidity chamber that can keep constant moisture and temperature. The qualitative characterization of the coupled stimuli of SME of goat hair fibers follows the similar approach and corresponding process in sequence [38]: (1) the effect of stimuli on the switch of hair fibers: ① under the set temperature of 90 °C and 65% of RH, the wrapped hair fibers were maintained for two hours to endow the hairs with full plasticization; ② the wrapped hair fibers were maintained in ethanol solution for full plasticization with polar chemical groups; ③ the wrapped hair fibers were illuminated under UV-light (315~400 nm, 300 mW/cm^2^) for two hours for a full interaction of UV-light and disulfide bonds; (2) the shape fixation ability of the hair fibers was observed: ① the hair fibers were then taken out of the chamber and cooled down to 20 °C naturally for one hour; ② the wrapped hair fibers were then taken out of the ethanol solution and dried naturally at room temperature; ③ the illumination of the UV-light on the hair fibers was stopped, and the wrapped hairs were maintained in darkness for one hour; then the wrapped hairs were stripped from the bar for observation of the temporary shape. The shape of the fibers in each process, including the temporary shape for investigating the fixation ability and recovered shape for memory ability, were observed using a camera; (3) the effect of the coupled stimuli factor on the shape recovery of the fibers was observed: ①/②/③ the temporary spiral hairs encountered with water/water/reductant solution (NaHSO_3_, 1 mol/L) and the shape recovery behaviors were recorded respectively. 

### 2.3. Quantitative Characterization of Hair Coupled Stimuli of SME 

**Figure 2:**
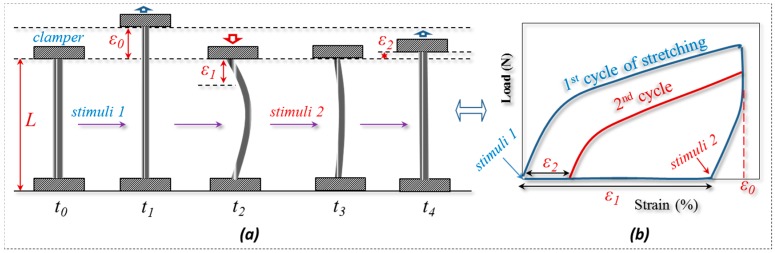
Stretching program of goat hair for quantitative characterization of the coupled shape memory effect (SME). (**a**) Five specific moments of the hair statuses in the program; (**b**) plot relationship of cyclic stretching load and strain of hair.

The coupled shape memory (SM) ability of α-keratin hair was studied quantitatively using four steps in one cycle of the stretching program, as shown in Figure 2. Instead of spiral shape fixation, and different from our previous paper on the multi-responsivity of four single stimuli of animal hairs [25], the variation of recovery degree from stretched hair to the original length under coupled stimuli is used for investigating the SME of α-keratin hair fibers. In Figure 2a, the original length (*L*) of goat hair after a particular environment in stimulus-1 was stretched up to a strain ($\varepsilon_{0}$), where the $\varepsilon_{0}$ is in the fiber plastic tensile region. Then stimulus-1 was removed and an opposite stimulus factor was exerted on the stretched fiber to fix the deformed shape at the moment of *t*_1_, and the gauge length returned to the original position at the moment of *t*_2_. Apart from the instant recovery of strain due to the elasticity, most of the tensile strain is observed unrecovered as $\varepsilon_{1}$ ($\varepsilon_{1}$ < $\varepsilon_{0}$). Macroscopically, the stretched hair is curved, as schematically illustrated at the moment of *t*_2_. At *t*_3_, the stretched fiber was under the coupled stimulus-2 normally; the unrecovered strain ($\varepsilon_{1}$) was decreased to a value that assumes to be $\varepsilon_{2}$. The onset of the 2nd tensile was from $\varepsilon_{2}$ and stretching to $\varepsilon_{0}$ at the second cyclic tensile. Accordingly, Figure 2b demonstrates the cyclic tensile program based on Figure 2a that can interpret the specific SM ability under coupled stimuli. Herein, stimulus-1 and stimulus-2 are responsible for unlocking the switch of keratin fiber to increase the mobility of macromolecule chains. The coupled stimuli are different factors but can be exchangeable in order to act on the keratin fiber. From Figure 2, there are two variables that thus come across for justifying the fiber coupled SM ability, that are $R_{f}$ (Equation (1)) and $R_{r}$ (Equation (2)), representing the shape fixation and recovery abilities respectively:

(1) $$R_{f}=\frac{\varepsilon_{1}}{\varepsilon_{0}}\times 100\%$$

(2) $$R_{r}=\frac{\varepsilon_{1}-\varepsilon_{2}}{\varepsilon_{0}}\times 100\%$$

This approach can refer to our work [25]. The ideal shape recovery shows a limit of “$\varepsilon_{2}$→0” after penetration and the removal of external coupled stimuli into and out of the fibers. Physically, a greater $R_{f}$ value means a higher sensitive switch to be on and off, whereas a higher value of $R_{r}$ implies a better shape recovery ability of the fiber. During the SME investigation of each fresh hair, the values of $\varepsilon_{1}$ and $\varepsilon_{2}$ are required to be tested ten times average values. The related $R_{f}$ and $R_{r}$ values are calculated with standard derivations.

### 2.4. Molecule Network Characterization of Hair Fiber during SME

The cross-section morphologies of goat hair fibers were gold-coated and observed using an environmental scanning electron microscope (ESEM, JEOL Model JSM-6490, Tokyo, Japan) under coupled stimuli (two hours of stimuli) conditions. Over the SME characterization, the chemical functional groups and intermolecular bonds of fibers under coupled stimuli were examined using Fourier Transform Infrared Spectroscopy (PerkinElmer Spectrum 100, FTIR Spectrometer, Waltham, MA, USA) in the range of wavenumbers between 4000 and 650 cm^−1^ using the ATR (Attenuated-Total-Reflectance) method. The absorption spectra were recorded with eight scans at a resolution of 16 cm^−1^. The angle (ϕ) of incidence light was adjusted to 39°, the ATR crystal was diamond (refractive index $n_{1}$ is 2.4), and the refractive index of the hair fiber ($n_{2}$) was around 1.5. The characterized depth of penetration ($d_{p}$) reaches up to 15 μm, which can fully reflect the internal chemical groups of hair cortex. Raman spectra yield similar but complementary information to the ATR-IR spectroscopy. It relies on Raman scattering from a laser light in the near infrared range. The light interacts with molecular vibrations, resulting in the energy of laser photons being shifted up and down. The chemical cross-links such as the disulfide bonds (DBs) of fibers under the SME investigation were characterized by a Horiba Jobin Yvon HR800 Raman spectrometer (HORIBA Scientific, Edison, NJ, USA), which was equipped with an *Ar* laser (λ = 448 nm, 180 mW) as the excitation light source and an Olympus BX41 microscope. The crystallinity of fibers under coupled stimuli conditions were determined by a Rigaku Smart Lab XRD system (9 KW, Rigaku, Tokyo, Japan) that is equipped with Cu/Kα radiation with a wavelength of 1.54 $\dot{A}$. The hair fibers were minced in the format of short chips (powder format) to cover the stage. The test 2θ range is from 5° to 30° and recorded at a scan speed of 10°·min^−1^ at 40 kV and 40 mA. On the other hand, the crystalline phase can refract a bright shinning spot based on Bragg’s law, while the amorphous area with irregular molecules gives dark background under a birefringence microscope due to light-wave phase cancellation. Here, a polarizing microscope (BD-XPL 3230, Boshida, Shenzhen, China) is applied to characterize the variation of the crystalline phase of the hair fiber under different processed states along the hair fiber axis.

## 3. Results and Discussions

### 3.1. Coupled Stimuli of SM Behaviors of α-Keratin Hair Fiber

**Figure 3:**
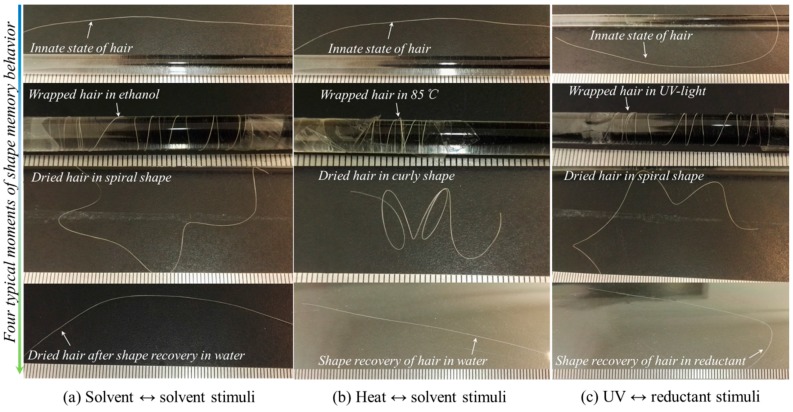
Shape memory behaviors of single goat hair fiber from groups of coupled stimuli of (**a**) ethanol-water; (**b**) heat-water; (**c**) UV-NaHSO_3_ solution (1 M/L).

Figure 3 demonstrates the results of three groups of coupled stimuli acting on the shape fixation and recovery behaviors of a single goat hair fiber. After stimulus-1 on the entangled shape fixation was applied, all goat hairs showed a curly or spiral shape that was maintained from their innate state. In comparison with temporary shape fixation, the stimulus of heat seems to result in better fixation ability with a denser helical state than that from stimuli of ethanol and UV-light, indicating that the heat stimulus opens the switch of hair fiber from the original state and closes the switch at the new dislocated polymer chains more easily than ethanol and UV-light. When the temporarily deformed hairs encounter stimulus-2, the fixed spiral shape of all hairs recover the innate shape completely, as shown the final state of hairs in each solution in Figure 3. However, it is noted that water and reductant solution both recover the innate shape easier than other stimuli, such as ethanol, heat, UV, etc. This means that small polar molecules work on the switch, which may be a hydrogen bond (HB) more efficiently, and this discovery enriches the types of stimulus in processing biopolymer fiber and helps us to select individual stimulus to finish the α-keratin hair fiber in shape setting.

**Figure 4:**
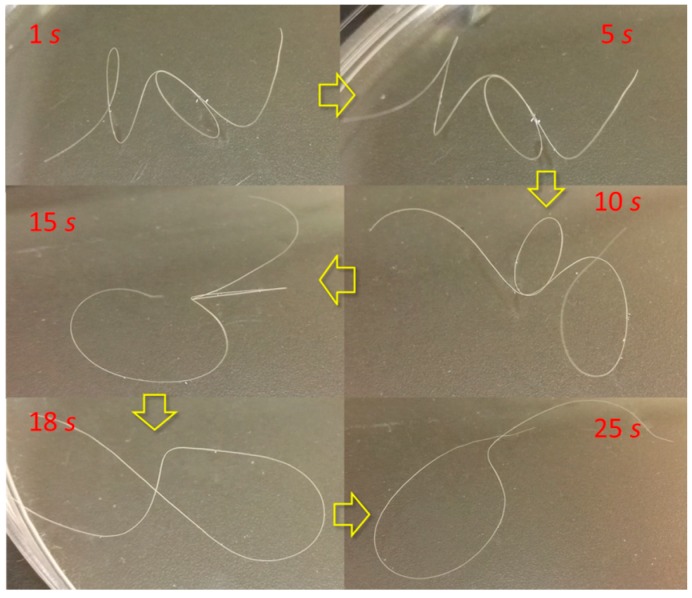
“Dancing” of temporary curly hair fiber on solvent for the hydration effect at different moments.

Regarding shape recovery behavior, Figure 4 demonstrates six moments of a goat guard hair with a temporarily curly shape “dancing” backward toward the innate shape since the deformed hair is dropped in water. The entire “dancing” process takes around half a minute. It is noted that the shape recovery gradually takes place from the part of hair fiber that encounters the water, which assumes that aqueous molecules switch on the HBs first at the deformed hair cortex in water. The energy of balance of temporarily deformed hair is disturbed so that the hair is dancing freely on the water in macroscale. The hair fiber looks to be “struggling” or “dancing” step by step. It is noted that the dancing duration of recovery using water or reductant solution is shorter than with ethanol, indicating that HBs are more sensitive to smaller polar molecules of aqueous molecules rather than ethanol molecules.

**Figure 5:**
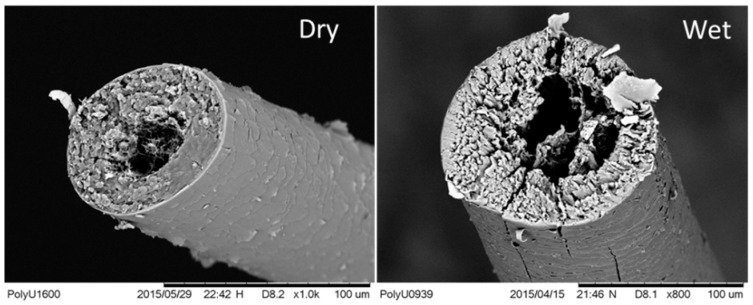
SEM images of cross-sections of goat hair fiber under dry and solvent-wet status.

Figure 5 compares a goat hair fiber under dry and wet conditions by SEM images of fiber cross sections. It is noted that dry goat hair is features a central cavity, namely the medulla, a surface scaly layer, and a main body layer between them called the cortex. An evident swelling is observed for the goat hair in water with the increased distance of macrofibrils. These fibrils in the hair cortex play key role in the hair coupled stimuli of SME, wherein the heating/UV-illumination process removes the free water and structural water between and inside the fibrils, which causes a decrease in the cortex size and more porous components in the morphology. Conversely, a wetting process to the heated/irradiated hair fiber penetrates a large amount of aqueous molecules or other polar molecular solvents into the space of the inter- and intra-fibrils, leading to an evident lateral swelling of hair cortex and a decrease of the hair medulla. The wetting process using water or reductant solution plasticizes the fibrils and enlarges the distance of two neighbor polypeptide chains by disrupting the HBs between the groups of N–H and C=O at the adjacent macromolecule branches. This enhances the mobility of the neighbor macromolecules, which acts as a stimulus for fiber shape recovery.

### 3.2. Quantification of Coupled Stimuli Sensitive SME Ability

Cyclic tensile to hair fiber is an effective way to manifest the SM ability by means of strain fixation and recovery under coupled stimuli to the clamped hair. The set tensile strain (0.1) is beyond the yield point of the hair (~0.03) so that the memory part can be identified from the elastic and permanent unrecovered strains. It should be noted that all the tests were under the same tensile speed, which means the recovery times are the same for each stimulus. The elastic strain is the instant recoverable strain released when the stretched hair starts to return to the original point, whereas the unrecovered strain can be found at the onset of the second tensile curve. The instant recovered strain from the original stretched hair is ascribed to the relaxation of the protein macromolecular chains under a stress-free state. A calculated $R_{r}$ value for each type of couple stimuli is nearly consistent with the qualitative characterization of goat hair, as shown in Figure 3a–c). For the heat stimulus for hair shape fixation, the stretching load of “2-load” shows a remarkably larger value than “1-load”, supporting the removal of water from the fibrils for stronger networks under conditions of tensile at 90 °C. 

**Figure 6:**
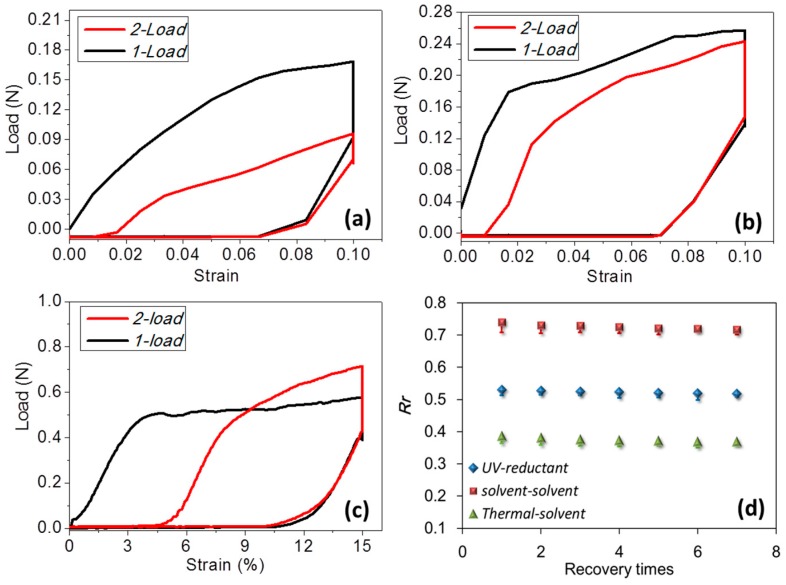
Experimental tests of the cyclic tensiles of goat hair fibers under the coupled stimuli of (**a**) UV (254 nm of light wave length)-reductant (NaHSO_3_ 1 M/L); (**b**) ethanol-water; (**c**) heat (85 °C)-water; and (**d**) variation of $R_{r}$ values from the stretched shape to the innate shape with the repetition of the SM program.

In detail, the shape fixation ratio (*R*_f_) reflects the ability of stimulus-1 to switch on the hair fiber and to lock the deformed hair in a temporary shape. Quantitatively, according to Equation (1) for Figure 6a–c, the calculated *R*_f_ values show that heat stimulus endows the hair with the highest fixed deformation, i.e., 0.73 of strain, in comparison with the fixed strain from UV of 0.68 and from ethanol of 0.70. This is ascribed to the increased number of HBs from the removal of free water after stretching and heating and assumes HBs to be one type of switch that benefits the locking of the tensile strain from less free water during heating. For the shape recovery ratio (*R*_r_) based on Equation (2), stimulus-2 (water) leads to the highest degree of shape recovery (0.74) based on the shape fixation using ethanol, followed with the reductant stimulus (0.53), corresponding to UV-light, and the water stimulus (0.39), corresponding to heating for shape fixation, respectively. $R_{r}$ correlates with the entropic stress resulting from netpoints and the connected network. The lowest $R_{r}$ value of the hair may imply the relative highest setting effect on the hair, indicating the number and type of netpoints for the recovery force may be the least. Water, in such a case, unlocks the most number of switch units, which causes the most recovery from the stress released from the netpoints. However, to different extents, the stimuli of heat, UV-radiation, and ethanol set the deformed shapes in different degrees, leaving variable recoveries stimulated by water. Herein, the stimulus of heat shows the most unrecovered strain, which indicates that heat may denaturate some α-keratin protein conformation, resulting in a little permanent setting of the hair fiber. Exposure of goat hair to UV-light can break down partial DBs (around 10% of the number of hair’s amino acids) and a reductant solution as the recovery stimulus can give rise to 53% of recovered strain. Water stimulus influences HBs, which causes a larger recovered strain, indicating HBs to much larger extent than DBs are the key switch in the coupled stimuli SME of hair fiber. 

Moreover, Figure 6d displays the stability of three groups of coupled stimuli for $R_{r}$ values in the seven cycles of SME tests, which indicates that the shape fixation and recovery abilities of animal hair are nearly invariable (decreasing less than 10%) and stable. This indicates the stable performance of coupled stimuli induced SME of α-keratin hair using heat, ethanol, and UV-light (under current experimental set factors) as the first stimuli for shape fixation and water and reductant solution as the second stimuli for shape recovery.

### 3.3. Characterization of Netpoint and Switch of SM Hairs

**Figure 7:**
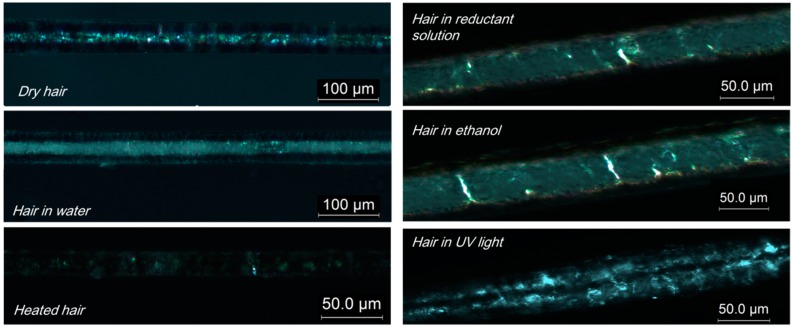
Images from a polarizing microscope that shows the discrete crystalline phase of different goat hairs under different states.

The variation of the internal structure of hair samples in six states have been evaluated from a few experimental aspects. Due to the stable structure of the crystalline phase of α-keratin hair fibers, its characterization is of importance to explore the related role in coupled stimuli of SM behaviors. Birefringence is an effective way to briefly know the variation of a crystal. For any animal hair sample, a regular intense array of macromolecules (crystalline phase) can refract a bright shinning spot according to Bragg’s law, while a disordered amorphous area with irregular molecules gives a dark background under a microscope due to light-wave phase cancellation. Figure 7 shows the representative images of a goat guard hair in six states under polarizing microscope. The images of hair under six such states can be obtained repeatedly using different samples. In Figure 7, all six images present many discontinuous shinning spots along the fiber axis, indicating the existence of a discrete crystalline phase in hair. Among these images, hairs in an original dry state and under UV-light show intense apparent shinning spots under birefringence, which means these states have the largest amount of crystals. The hairs in solvent (e.g., water, ethanol, etc.) environments display a relatively smaller number of spots (fewer crystals), which indicates that the margin of crystalline phase may be disrupted by polar solvents, and the hair after the heating process shows a remarkably decreased number spots, which means that heating causes the hair with the least crystals. This is the reason for the smallest $R_{r}$ value of the hair sample in the thermal-water stimuli of SME performance because the fewest crystals are left in hair after heating, in which crystal is supposed to be a netpoint to supply the released entropic force for shape recovery. 

**Figure 8:**
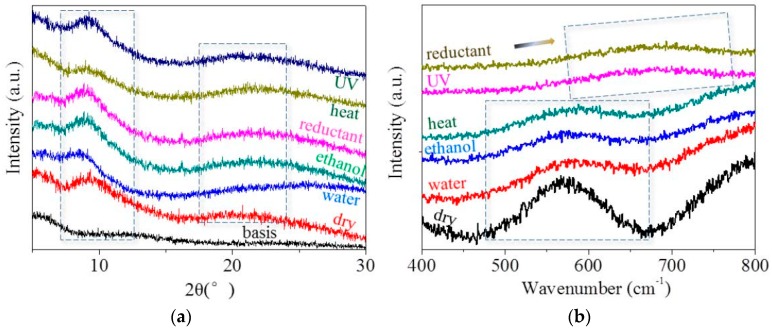
Structural component characterization of goat guard hairs under original (dry), wet (water), soaked in ethanol, reductant (NaHSO_3_, 1 M/L) solution, heat, and UV-radiation conditions: (**a**) XRD raw data of each hair state on the basis of baseline data; (**b**) Raman spectra for characterizing the disulfide bonds of goat hairs.

Consistent with the birefringence images that reflected the information on the crystalline phase, Figure 8a shows the XRD patterns of goat hairs in their original state and in the states after responding to five stimuli, which is also a relatively precise way to manifest the variation of the crystalline phase. The diffraction shoulders and peaks from the original state and arises by heating, reducing hydration and radiation with different sharp peaks, especially at the abscissa of 2θ = 9° (0.98 nm) and 22° (0.46 nm), indicating the strong characteristic α-helix and β-keratin crystalline phases, respectively. The identical intensities of both characteristic peaks at the states of original (dry) and UV-radiation (UV) indicate the invariable amount of a crystalline phase under such stimulus because the light-illumination process scarcely has an effect on the hair crystal. However, both characteristic peaks display slightly decreased intensity for the hair under the stimuli of water, ethanol, and reductant solution, indicating that the polar ions have a slightly destructive effect on the margin of the crystalline phase that is composed of dense HBs between inter-macromolecule chains. Coordinately with birefringence, the XRD characteristic peak (α-helix type) of goat hair after heating shows a remarkable decrease in intensity, indicating the partial destruction of the crystalline phase and the reason for the lowest recovery ability (*R*_r_ = 0.39) from the netpoints of crystals.

In comparison to goat hairs of the Raman spectra under coupled stimuli conditions, it is noted that the “dry”, “water”, “ethanol”, and “heat” curves can be viewed as nearly coincident in the Raman scanned regions (abscissa values of Figure 8b). Specifically, the symmetrical DB mode from 500 to 650 cm^−1^ can be found as a broad characteristic peak that is associated with several molecule conformations [39,40], i.e., *g*-*g*-*g* (510 cm^−1^), *g*-*g*-*t* (525 cm^−1^), and *t*-*g*-*t* (540 cm^−1^) (*g* and *t* denote *gauche* and *trans*) conformations. Figure 8b suggests that the present hydration (water and ethanol) and heating processes slightly break down a little proportion of DBs, leading to the intensity of the characteristic peak being lower than the original (dry). This means that the hydration and heating energy can enhance the spatial distance and the vibration of the covalent bond slightly; however, a drying or cooling process can reduce the vibration significantly to have the same intensity of Raman curve as the original. However, the abscissa of DB characteristic peaks are constant under four states, indicating that there is no conversion of the disulfide bond (–S–S–) and thiol group (–SH), and this chemical cross-linker –S–S– may act as a weak netpoint for hair’s water, heat, and ethanol related coupled SM behaviors. Nonetheless, hairs under UV-illumination show the Raman characteristic peak right shifting for the cause of conversion of –S–S– bond to –SH groups. An opposite process would lead to the left shifting of the characteristic peak. This indicates that DB may act as a switch of hair when responsive to such stimuli.

**Figure 9:**
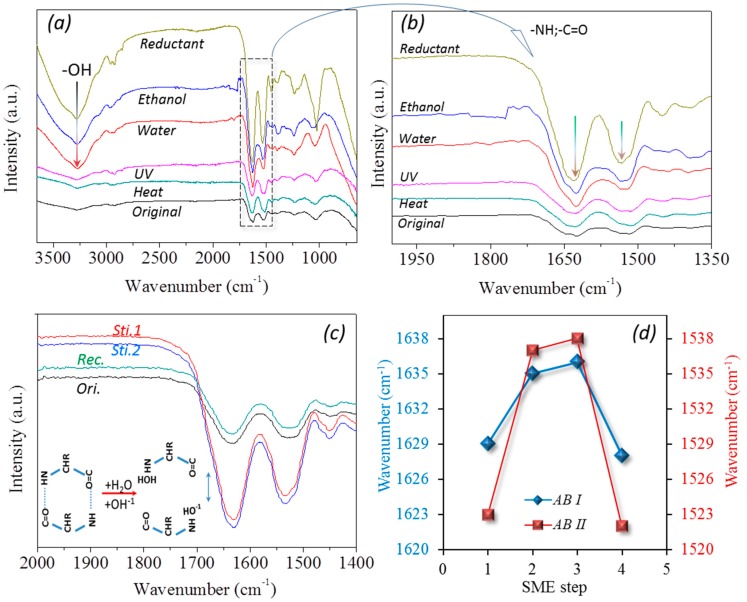
FTIR characteristic peaks of goat hair fibers under original status and processed states under heat, UV light, water, ethanol, and reductant solutions, where (**a**) shows the whole spectra and the dashed frame is presented in the enlarged figure; (**b**) for the specific functional groups –NH and –C=O representing HBs switched on and off, using wavenumber shifting and ratio variation of peak intensity that also means disruption and re-formation of HBs from IR peak shifting; (**c**) a case of goat hair under a group of coupled ethanol-water responsive key SME steps that (**d**) shows the wavenumber shifting of the two characteristic peaks at the four key steps.

Figure 9a,b shows six FTIR curves of the goat hairs under original status and processed states under heat, UV-light, water, ethanol, and reductant solutions. For the hair fibers soaked in the water, ethanol, and reductant solutions, a broad absorption band at around 3400 cm^−1^ is introduced to the original dry FTIR curve, corresponding to the free –OH^−1^ of the wet sample [41]. In particular, both the characteristic peaks of the C=O stretching (Amide band I) and N–H bending (Amide band II) vibrations are shifted to higher wavenumbers; from 1628 to 1636 cm^−1^ and 1522 to 1538 cm^−1^, respectively, as shown the dash frame in Figure 9a and the enlarged spectra of these two peaks in Figure 9b. This implies that the intermolecular HBs are formed between residues and aqueous/ethanol molecules during the hydration process [23,42]. Therefore, the absorbed small polar molecules within the biopolymer hair exist in the two distinct states of free water/–OH^−1^ and structural water.

Figure 9c gives four detailed FTIR curves for characterizing four key SM program steps in one case of coupled stimuli of SM behavior, where the original (Ori.) and shape recovered (Rec.) states share almost the same IR profile, and stimulus-1 (sti.1) and stimulus-2 (sti.2) display a similar IR profile. Consistent with the IR curves of “water” and “ethanol” in Figure 9b, it is noted that the intensity ratio of the characteristic peaks of N–H bending (Amide band II) to C=O stretching (Amide band I) vibrations are evidently different for the hair under original (Ori. and Rec.) and wet (water and ethanol) conditions. The wavenumber shifting and varied intensity ratio along with the key steps are calculated. In respect to wavenumber shifting, as shown in Figure 9d, the characteristic peak of C=O stretching is increased by 7 cm^−1^ from the original dry to stimulus-1 (ethanol, step 2) and stimulus-2 (water, step 3) states; in turn, the wavenumber is decreased by 8 cm^−1^ from the wet to the recovered state (step 3 to step 4). This indicates that the two-coupled stimuli have the same effect on the hair switch. Similarly, both bands manifest similar trends in the shifting of IR wavenumbers under other coupled stimuli, such as heat-water and UV-reductant. This reversible shifting related to the conversion between the original and recovered to stimulus-1 and stimulus-2 conditions suggests that the intermolecular HBs undergo reversible destruction and formation processes, accordingly [43].

Furthermore, the intensity ratio between the two characteristic peaks (Amide bands I and II) of hair shows variations from the hair states of original dry (97.87%) to wet (sti.-1 = 85.45%) to wet (sti.-2 = 85.20%) to recovered dry (98.72%) for the coupled stimuli of ethanol-water, from the original dry (97.87%) to UV (sti.-1 = 97.92%) to wet (Reductant = 84.6%) to recovered dry (98.64%) for the coupled stimuli of the UV-light-reductant solution, and from the original dry (97.87%) to heat (sti.-1 = 99.42%) to wet (water = 84.6%) to recovered dry (98.64%) for the coupled stimuli of the heat-water solution. When the hair fiber is dried so that the peak intensity ratio almost equals one, it means that the intensities of the characteristic peaks at 1620~1640 cm^−1^ and 1510~1535 cm^−1^ show the same amount of carbonyl groups (C=O…) and imino groups (N–H…). At the recovered dry state, this indicates that the excess of free aqueous molecules has been removed from the hair shaft during the drying process. When the hair fiber is in a wet state, the penetrated small polar molecules can disrupt the HBs formed between carbonyl and imino groups. As shown in the inset of Figure 9c, under this dynamic state, each aqueous molecule or other polar solvent molecule was attached to each imino group for the polar attraction between the atoms of hydrogen (N–H) and oxygen (H–O–H). This leads to a decreased number of discrete amino groups for 1510~1535 cm^−1^ and a remarkably reduced intensity ratio between the two characteristic groups. This wetting process of hair switches on the HBs, locking the shape under the free state, and recovers the innate shape with the aid of netpoints. However, it should be pointed out that the interaction of polar molecules between the keratin macromolecule chains only demonstrates the transformation process of each coupled SME step, while the interaction dynamics (speed of switch on and off in macroscopic) are not involved in the illustration. 

**Figure 10:**
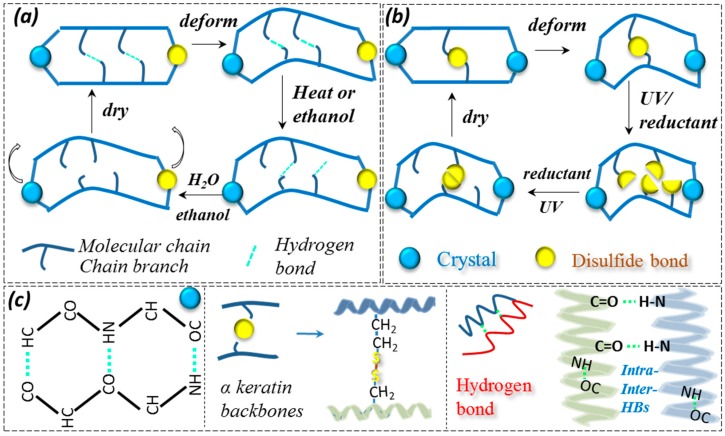
The mechanism illustration of coupled stimuli responsive SME of α-keratin hair fiber: (**a**) crystal and disulfide bonds acting as netpoints and hydrogen bonds acting as switches for the SME of hair fiber under the coupled stimuli of heat-water and ethanol-water, separately; (**b**) crystal and disulfide bonds acting as netpoints and switches for the SME of hair fiber under the stimuli of UV (reductant) and reductant (UV), reversibly; (**c**) symbols of crystal made of dense hydrogen bonds, disulfide bonds between two keratin backbones, and hydrogen bonds at intra-macromolecules and between inter-molecules chains.

Figure 10 shows two types of coupled stimuli of the SME mechanism; namely, HB and DB as the switch, respectively, the real corresponding chemical structure (Figure 10c) for the crystal, and two types of bonds. For HB (hydrogen bonds at intra- and inter- molecule chains) acting as a switch from a molecule motion viewpoint, a schematic illustration is proposed to correspond to the four steps of coupled stimuli of a solvent-solvent/thermal-solvent sensitive SM program. Here, crystals (dense HBs between regular aligned molecule chains) and the DBs (a covalent bond between macromolecule branches) are assumed as the netpoints and the HBs as the switch. The SMP network is deformed into a temporary shape with stored internal stress. A heating or hydration (ethanol/water, stimulus-1) process is applied that can release the stress from the HBs, and new HBs are formed at new dislocated positions of macromolecule chains. Another hydration (ethanol/water) effect takes place when the deformed hair is responsive to stimulus-2 (ethanol/water). The switch is open again and the entropic stress from the netpoints brings the deformation back to the innate shape, which gives a cycle of the SM program. For DBs acting as a switch, as shown in Figure 10b, the crystal is supposed to be a netpoint, and only the external stimulus can switch on and off the DBs that could form the coupled SME. Experimental data indicates that coupled UV-reductant solution endows the goat hair fiber with good SM behaviors. It should be noted that the coupled stimuli can be reversible in sequence. Take HB as switch, for example; stimulus-1 can be water, and stimulus-2, then, is ethanol. The difference that should be concerned is the completion duration of a whole SM cycle; the recovery time determines the SM program duration. Furthermore, it is found that the stronger and more number of netpoints in the hair would give rise to better coupled stimuli induced SME, which may inspire researchers to design more remarkable and rapid-reactive SM of synthetic polymers.

## 4. Conclusions

In this work, α-keratin hairs were investigated in conjunction with three types of coupled stimuli responsive SM behavior and related mechanisms in molecular and structural networks. It is found that the innate shape of a natural goat hair can be recovered through hydration and reducing effects after a deformed shape is fixed using heating, hydration, and UV-radiation. Tested shape fixation, recovery, and repeatable memory cycles of typical goat hairs demonstrated that α-keratin hairs are good SM materials. The exploration into structural components and chemical bonds (crystal, DBs, and HBs) reveals a SM structural network model in macromolecular assemblies of such smart hair fibers. Thereafter, a hybrid network consisting of single-switch (HB or DB) and twin-netpoint (crystal and DB/single netpoint crystal) is identified. It is inferred that more remarkable SME of a hair is due to stronger netpoints in the biopolymer networks in which crystals and DBs can work together or separately while HBs act as a switch. This mechanism varies in different hair types of α-keratin biopolymers and realizes different levels of SM ability. This study is expected to provide inspirations for developing more realistic, remarkable, and rapid-responsive SMPs.
